# Work together, move together—Cooperation and rapport promote interpersonal synchrony

**DOI:** 10.1371/journal.pone.0333709

**Published:** 2025-10-22

**Authors:** Clara Scheer, Daniel L. Bowling, Niklas A. Hungerländer, W. Tecumseh Fitch, Lisa Horn

**Affiliations:** Department of Cognitive Biology, Faculty of Life Sciences, University of Vienna, Vienna, Austria; University of Missouri Columbia, UNITED STATES OF AMERICA

## Abstract

Human movement synchrony is one fundamental driving force for social bonding and yet not all individuals are equally likely to engage in interpersonal synchrony. Movement synchrony increases collaboration and feelings of rapport, but it remains unclear whether cooperative interactions between two individuals also result in greater interpersonal synchrony. The aim of the current study was to investigate whether cooperating in one task increased spontaneous movement synchrony in a subsequent unrelated task. Thirty-four same-gender dyads first worked on a jigsaw puzzle – either collaboratively or individually – and subsequently jumped on trampolines facing each other. Additionally, we investigated the potential effects of the participants’ rapport and mood during the experiment. As predicted, dyads who had worked on the puzzle collaboratively displayed greater movement synchrony when later jumping on trampolines than dyads who had worked individually. When we tested for the additional influence of the participants’ initial rapport and mood, however, the effect of the collaborative treatment was qualified by a stronger effect of initial rapport. The results of the current study provide evidence that collaborative interactions and initial rapport are important stepping stones towards interpersonal synchrony and add to the growing body of evidence suggesting that human cooperation and interpersonal synchrony are essentially connected.

## Introduction

One fascinating aspect of human interactions is our tendency to synchronize our movements with those of an interaction partner [1,2]. This can be as trivial as two individuals unconsciously synchronizing their strides while walking side-by-side [3], or an enthusiastic audience clapping in synchrony after an entertaining theater performance. Humans engage in interpersonal synchrony automatically and unconsciously [4]. The underlying mechanism of this phenomenon is a self-organized process which builds on the same lawful principles governing other coupled oscillators, namely to synchronize over time [5]. The maintenance of interpersonal synchrony likely depends on predicting and monitoring a partner’s actions [6]. Beyond that, synchrony is also a fundamental driving force for social bonding in humans [1,6] – so much so that it has repeatedly been referred to as a form of ‘social glue’ [7]. From an evolutionary point of view, the link between interpersonal synchrony, defined here as movements coordinated in time and form [1], and social bonding is deeply rooted in human history. Moving in synchrony increases feelings of unity when participating in collective rituals [8], it enhances a sense of similarity and connectedness [9], and promotes self-other merging [10]. In addition, the notion exists that this connection can also emerge in the opposite direction: Hagen and Bryant [11] theorize that a group’s inherent collaborative quality influences the degree to which this group is able to perform synchronous displays, such as group dances. Therefore, a well synchronized dance provides information about the group’s ability to act collectively for mutual goals [11,12]. While the success of a choreographed dance or a professional music ensemble may not require a supportive predisposition of the interacting individuals [13], spontaneously synchronized movements, like stepping in place [14], or arm movements [15], are likely enhanced by a cooperative attitude and mutual connection, as described by Hagen and Bryant [11]. Accordingly, the phenomenon that moving in synchrony increases feelings of unison and, at the same time, a certain degree of cooperative aptitude and rapport might be needed for individuals to spontaneously synchronize warrants thorough examination.

There is empirical evidence to support both notions. On the one hand, experimentally induced synchrony has been shown to increase prosocial attitudes and behaviors, such as perception of similarity, compassion, and collaboration. For example, participants walking in step feel more similar to each other than participants who do not engage in a synchronous task [16], and individuals tapping in synchrony show a stronger sense of similarity, as well as more compassion and altruistic behavior than individuals tapping asynchronously [17]. Participants who are told to move in synchrony are also more successful in subsequently coordinating their movements in a joint action task [9] and finding a solution in a verbal collaborative problem-solving task [18]. Conversely, participants’ positive orientations toward others and initial levels of rapport between two individuals have been found to enhance interpersonal synchrony. For instance, prosocially-oriented individuals are more likely to synchronize arm movements than proself-oriented participants [15], and individuals are more likely to engage in synchrony with people they like [19], people they find attractive [19], and those who made a positive first impression [3,14]. However, the agreeableness of a confederate does not appear to influence the synchrony of hand movements in a rhythmic task [13]. These studies demonstrate that movement synchrony increases rapport and collaboration. But in line with Hagen and Bryant’s hypothesis [11] and given the inconsistencies of previous research, it would be crucial to test whether previous cooperative interactions also induce greater interpersonal synchrony.

Existing evidence suggests that participating in a collaborative task increases movement coordination and physiological synchrony during the task [20]. For example, the postural sway patterns of two individuals synchronize during a cooperative verbal task, even without visual contact, suggesting that verbal cooperation alone can induce bodily synchrony [21]. However, the degree of movement synchrony depends on the structure of the task, such as the specific instructions (e.g., free collaboration vs. assigned leader/follower roles [22]) or the tone of a conversation (e.g., friendly conversations vs. arguments [23]. Spontaneous synchrony of gestural movements emerges while working jointly on a verbal problem-solving task [18]. Greater coordination during the task also predicts more success in solving the task, but only when participants experience no synchronous or asynchronous interactions in a preceding movement task [18,22]. Allsop et al. [24] found that pairs of participants who had to move balls from one container to another synchronized their movements more when they were instructed to cooperate than when they were instructed to compete in the task, particularly when the task was difficult. Participants in the cooperative setting were also more successful than participants in the competitive setting [24]. When looking for effects that endure beyond the collaborative task itself, Yun et al., [25] found that participants who had practiced following the finger movements of a partner exhibited more synchronous finger movements and brain synchrony measured via EEG hyperscanning in a subsequent free interaction phase in which they were not instructed to follow the other’s finger movements anymore. While the above-mentioned studies indicate that cooperative tasks elicit greater interpersonal synchrony, no studies to date have investigated whether cooperating in one task increases spontaneous movement synchrony in a subsequent, unrelated task.

Therefore, the aim of the current study was to test whether participant dyads who collaboratively worked on a jigsaw puzzle exhibited more synchrony when later jumping on trampolines than participant dyads that worked on the jigsaw puzzle individually. We further investigated whether the participants’ initial rapport and mood influenced jumping synchrony and whether working collaboratively or individually produced any changes in rapport and mood from the beginning to the end of the experiment. We predicted that collaboratively working on the jigsaw puzzle would induce greater jumping synchrony than working on the puzzle individually and that rapport, but not mood, would also positively influence synchrony. Further, we hypothesized that both rapport and mood would increase more after working on the puzzle collaboratively than individually.

## Materials and methods

### Participants

We recruited sixty-eight participants in this study (34 female, 34 male; participant age: **M* *= 25.69 years, *SD *= 5.32, min = 18, max = 40; see section “data preparation and analysis” for details about sample size estimation). We formed participant dyads by scheduling one person via the online research participation system of the Department of Cognitive Biology and inviting a second person from a pool of potential participants recruited via advertisements in public places (e.g., at the university). By doing so, 34 same-gender dyads with no familiarity or relationship prior to participation were formed. Upon arrival, we confirmed that there was no familiarity between the participants by verbally asking them whether they already knew each other. Prior to the experiment, written informed consent was obtained from all participants and participation was voluntary. After the experiment, participants were debriefed and received financial compensation for their time and effort. All procedures were carried out in accordance with the Declaration of Helsinki and had been approved by the ethics committee of the University of Vienna (Ref. No. 00063). All data were collected between 24/04/2014 and 07/08/2014.

### Experimental design

The experiment consisted of a treatment phase in which the participants worked on a jigsaw puzzle and a test phase in which the participants jumped on separate trampolines. Participant dyads were randomly assigned to one of two experimental groups. In the treatment phase, dyads in the group *together* worked on one jigsaw puzzle together, whereas participants in the group *separate* worked individually on one jigsaw puzzle each. The test phase was the same for both experimental groups. The participants of each dyad jumped on trampolines while facing each other.

Additionally, the participants filled out two questionnaires, one prior to the treatment phase and one after the test phase. The two questionnaires contained the same questions about the participants’ mood [26] and their perceived rapport [27] with the dyad partner.

### Experimental materials and set-up

The introduction, treatment phase, and debriefing took place in a large, quiet room. The room contained two desks and chairs positioned at opposite sides of the room. An additional desk was placed in the center of the room for the treatment phase of the group *together*. We used commercial jigsaw puzzles that consisted of 100 pieces (49 cm x 36 cm, “The Animal Friends”, ©Ravensburger, ©Disney) in the treatment phase.

The test phase took place in a separate, quiet room. The room contained two trampolines that were placed in the center of the room, 80 cm apart from each other. The trampolines differed in size (trampoline A: diameter = 100 cm, trampoline B: diameter = 80 cm). The size difference resulted in different tension of the jumping nets and thus in differences in comfortable jumping height and frequency between the two trampolines. There were two colorful pictures with schematic drawings of jigsaw puzzles (26 cm x 21 cm) on the walls behind the two trampolines. Next to the trampolines, at about 1 m distance from either trampoline, was a desk that contained technical equipment.

A belt with an attached accelerometer was fixed around each participant’s waist to measure jumping frequency. We used small 3-axis accelerometers (PhidgetSpatial 0/0/3 High Resolution, 3 cm x 4 cm x 0.5 cm, http://www.phidgets.com/products.php?product_id=1043). The accelerometers were connected via USB cables (length for each participant: 195 cm) to a Mac mini positioned on the desk next to the trampolines. Data were recorded as a text file with a custom software programmed in Python v2.6 by Jinook Oh. We used two cameras (Canon Legria FS306) to record the participants’ behavior during the experiment. We assessed any irregularities and compliance with the experimenter’s instructions from the video recordings after the experiment. After this assessment, the video recordings were deleted.

### Questionnaires

We used a questionnaire to assess each participant’s mood (shortened version of the Positive and Negative Affect Scale, PANAS [26] and rapport with their dyad partner (shortened version of a standard rapport questionnaire [27]) before and after the experiment. Each part consisted of three positive and three negative adjectives (mood: *happy*, *social*, *motivated*, *depressed*, *inactive*, *shy*; rapport: *pleasant*, *harmonious*, *satisfying*, *embarrassing*, *boring*, *cold*). Participants indicated how much each of these adjectives corresponded with their current mood and with how they anticipated or experienced the interaction with their dyad partner on a continuous graphical scale ranging from *not at all* to *completely*. Internal consistency was good for mood and rapport at both measurement times (Cronbach’s α ≥ 0.77 across both traits and measurement times). We then reversed the negative items and calculated averages across all items of each trait per measurement time.

### Procedures

Upon arrival, participants were welcomed, introduced to each other, and informed about the experiment. The procedure, from greeting participants and explaining the study to guiding them through the questionnaire, was designed to create a relaxed, welcoming environment and eliminate awkward silences between the participants. The cover story framed the experiment as an investigation into the relationship between cognition and movement, with the jigsaw puzzle assessing cognitive engagement and the trampoline jumping assessing movement. Thereafter, participants gave written informed consent. Immediately prior to the treatment phase, the participants were seated back-to-back at the desks on opposite sides of the room and asked to complete the first questionnaire about their mood and expected rapport with their dyad partner. Because we aimed to compare the participants’ expected rapport with the actual rapport felt after the interaction, we asked them at this point of the experiment to evaluate their baseline expected rapport toward each other.

In the treatment phase, participants in the *separate* group remained seated back-to-back at the desks on opposite sides and were instructed to work individually, each on one jigsaw puzzle for ten minutes. Conversely, participants in the group *together* group were moved to one desk in the center of the room, where they sat facing each other and were instructed to work on one jigsaw puzzle together for ten minutes. Participants in both experimental groups were told to work at their own pace and that there was no requirement to finish the puzzle. Importantly, no specific instructions were given to participants about the collaborative or individual nature of the treatment phase in either group. To avoid an atmosphere of surveillance, the experimenter left the room during the treatment phase.

The treatment phase was directly followed by a test phase. The experimenter led the participants into a different room in which the two trampolines were positioned. The participants were asked to stand on one trampoline each and instructed to start jumping at a verbal signal from the experimenter and continue jumping at a speed that was comfortable for them for two minutes. The participants jumped facing each other, but were instructed to focus on the colorful picture on the wall behind their partner throughout the two minutes of jumping. This instruction served to mitigate explicit attempts to synchronize and to conceal the experiment’s research question, while also limiting potential awkward feelings that might have arisen from excessive eye contact in this unconventional situation. To further limit potential social discomfort, the experimenter left the room during the test phase after signaling for jumping to begin. Nevertheless, signs of social discomfort, such as giggles, facial expressions, or verbal remarks, as well as compliance with the experimenter’s instructions, were evaluated in video recordings after the experiment in consideration of potential differences between groups.

After the test phase, the participants returned to the first room and were again seated back-to-back at the desks on opposite sides of the room to provide a post-test rating of their mood and perceived rapport with their partners. Additionally, this second questionnaire asked about the participants´ experience with jigsaw puzzles, trampoline jumping, their level of expertise in music and dancing, and their assumptions about the study’s purpose. After the experiment, the participants were debriefed and paid for their time.

### Data preparation and analysis

We conducted a sample size estimation with G*power [28]. Effect size was expected to be large based on previous studies on interpersonal synchrony (e.g., [24,29,30]). The sample size estimation revealed a sample size of 36 dyads for detecting large effects in our most complex model (*f*^2^ = 0.35, 1-β = 0.80, α = 0.05). We recruited a sample close to this estimated sample size (**N* *= 34 dyads). No additional data were collected after the initial data analysis.

Video assessment of the participants’ behavior revealed no marked issues with social discomfort, suggesting that the experimenters were successful in creating a friendly and comfortable atmosphere for this study. However, the videos did reveal issues with the data collected from two participants (both male): one participant lost the accelerometer during the test phase and one participant engaged in behavior that differed strongly from the experimenter’s instructions (i.e., he later revealed that he suspected the purpose of the study and intentionally desynchronized from his partner’s jumping movements by doing jumping jacks on the trampoline). The data of the two dyads that included these two participants were therefore excluded from subsequent analyses of experimental effects on synchrony, resulting in a final sample size of **n* *= 32 dyads (group *together* = 17 dyads, group *separate* = 15 dyads) in these analyses.

Data preparation of the jumping data was performed in Matlab 2010b. For each dyad, a synchrony score was computed by comparing accelerometry data between participants, specifically in the y-axis, representing the vertical acceleration of each participant as they jumped on the trampoline. Prior to comparison, four preprocessing steps were applied to each individual’s jumping data. First, the initial and final 10 s of each 120-second recording were removed to exclude potential differences in starting and stopping. Second, gaps in the data (i.e., timestamps with no data due to accelerometry signal loss) were identified and repaired by linear interpolation (function ‘interp1q.m’ [31]). The number of gaps per dyad ranged from 0–25 (*Mdn* = 1); gap duration ranged from 8 to 330 ms (*M* = 0.142 ms, *SD* = 0.111). Third, the gap-repaired data were low-pass filtered at 2 Hz to remove variation unrelated to the primary jumping motion (3rd-order Butterworth filter, designed and implemented with functions ‘butter.m’ and ‘filtfilt.m’ [31]). Fourth, the mean of each filtered data trace was subtracted from itself, bringing recorded values into oscillation around zero. Synchrony between participants— here defined as jumping together in time with the same period and phase—was assessed by calculating the normalized cross-correlation coefficient at zero-time lag (function ‘xcorr.m’ [31]). This value represents the degree of alignment between participants’ jumping data in line with the above definition (i.e., co-occurring movements with shared period and phase) rather than other forms of temporal coordination (e.g., phase-locking at a non-zero angle). While literature on dynamical systems more broadly defines synchrony as stable phase locking irrespective of the specific lag between two actions [32], we focused specifically on zero-lag synchrony, as it most directly reflects our hypothesis regarding simultaneous, temporally matched trampoline jumping. Note that this method of assessing synchrony does not require perfect overlap to yield a value proportional to the degree of temporal alignment between participants. Instead, the value of the cross-correlation coefficient, which is at a maximum of 1 for perfect alignment, gradually decreases as relative phase angle increases, reaching a minimum of −1 at 180˚, before increasing to 1 again as the signals realign in the next cycle. Accordingly, participants who are more synchronized (i.e., jumping at nearly the same times) will have higher scores than those who are less synchronized. The values of the zero-lag cross-correlation coefficient for each dyad were normalized by scaling each participant’s individual jumping function so that its autocorrelation at zero lag equaled 1, prior to determining the cross-correlation coefficient.

As a complementary visualization of participants’ synchrony over time, we plotted time-series plots for the two dyads with the highest jumping synchrony scores and the two dyads with the lowest scores. These plots illustrate the underlying sinusoidal movement patterns, as well as the presence (or absence) of consistent phase relationships over time. Additionally, we generated Cross-Recurrence Plots (CRPs) to visualize cross-recurrence patterns of the jumping behavior, and complemented these visualizations with metrics derived from Cross-Recurrence Quantification Analysis (CRQA). The CRQA allows to capture the complex, dynamic, and often non-linear coordination that emerges in social interactions. It has been successfully applied in studies of conversation [33], postural sway [21], and joint action [34], providing valuable insights into interpersonal coordination [35]. We computed window-based CRPs to illustrate recurring coordination structures, thereby capturing typical patterns across the trial rather than moment-to-moment changes. For each dyad, the vertical jumping trajectories were segmented into ten-second windows, moving across the signal in five-second steps, resulting in 18 windows per dyad. Within each window, CRPs were calculated in reconstructed phase space, using a time delay (τ) of 1, determined as the first lag where the autocorrelation dropped below 0.1 [36], and an embedding dimension (m) of 2, identified via the False Nearest Neighbors method with a threshold of <1% false neighbors [37]. The recurrence threshold (∊) was set to a fixed value of 0.4, corresponding to 10% of the maximum phase-space diameter (Dmax). A fixed radius ensures symmetry of the recurrence matrix and a consistent neighborhood size, which is particularly suitable for regular, periodic dynamics such as trampoline jumping [36]. Distances were quantified using the Euclidean metric. CRPs were then averaged across windows and dyads for each condition (*together* vs. *separate*), producing condition-specific CRPs that reflect the presence of recurring synchrony patterns — where diagonal structures indicate periods of close temporal alignment. The cross-recurrence plot (CRP) is constructed by converting the pairwise distance matrix D into a binary matrix, where entries are set to 1 if the distance is less than or equal to the threshold epsilon and 0 when it exceeds this threshold, indicating recurrence of similar states between the two signals. To complement the visual interpretation of the CRPs, we additionally computed key CRQA metrics—Recurrence Rate (RR), Determinism (DET), average diagonal line length (L), and Laminarity (Lam).

Statistical analyses were performed in R 4.3.1 using RStudio 2023.06.1, using *rstatix* (version 0.7.2). In a first step, we used two-sample Student’s *t*-tests to check for gender differences in our variables of interest—jumping synchrony, rapport, and mood—before and after the experiment. None of the variables differed significantly between male and female participants (**p* *> 0.05). Consequently, we did not include gender as a factor in the following analysis.

To test our hypothesis that collaboratively working on the jigsaw puzzle would induce greater jumping synchrony than working on the puzzle individually we used a two-sample Student’s *t*-test to compare the jumping synchrony score of the experimental groups, *together* and *separate*. To further investigate whether initial mood and rapport also affected jumping synchrony, we calculated a linear model with the dyad’s jumping synchrony score as the dependent variable and *experimental group* (together vs. separate), average *initial rapport*, and average *initial mood* (i.e., the rapport and mood scores reported at the beginning of the experiment, averaged across the two members of each dyad), as predictors. The dependent variable jumping synchrony score was bounded between −1 and 1, violating the assumption of unbounded data given for linear regressions. Therefore, we applied Fisher’s *z*-transformation (*z* = atanh(*r*)) to the jumping synchrony score prior to including it as a dependent variable in the linear model. For interpretability, we back-transformed the results (*r* = tanh(*z*)) to the original scale of the dependent variable (bounded between −1 and 1) where relevant. Finally, to test whether rapport and mood increased more after working on the puzzle collaboratively than individually, we calculated two mixed ANOVAs (type III; package *rstatix* [38,39] with rapport or mood as dependent variables, respectively, and *experimental group* (together vs. separate; between-subject), *measurement time* (before vs. after, within-subject), and their interaction as predictors. Planned post-hoc comparisons were performed with paired *t*-tests with Bonferroni correction. For *t*-tests and ANOVAs, we confirmed that assumptions for conducting the tests were met (normal distribution, homoscedasticity). Adequacy of the linear model was confirmed by assessing normal distribution of residuals with a Shapiro-Wilk test and by visually inspecting Q-Q plots.

## Results

**Fig 1:**
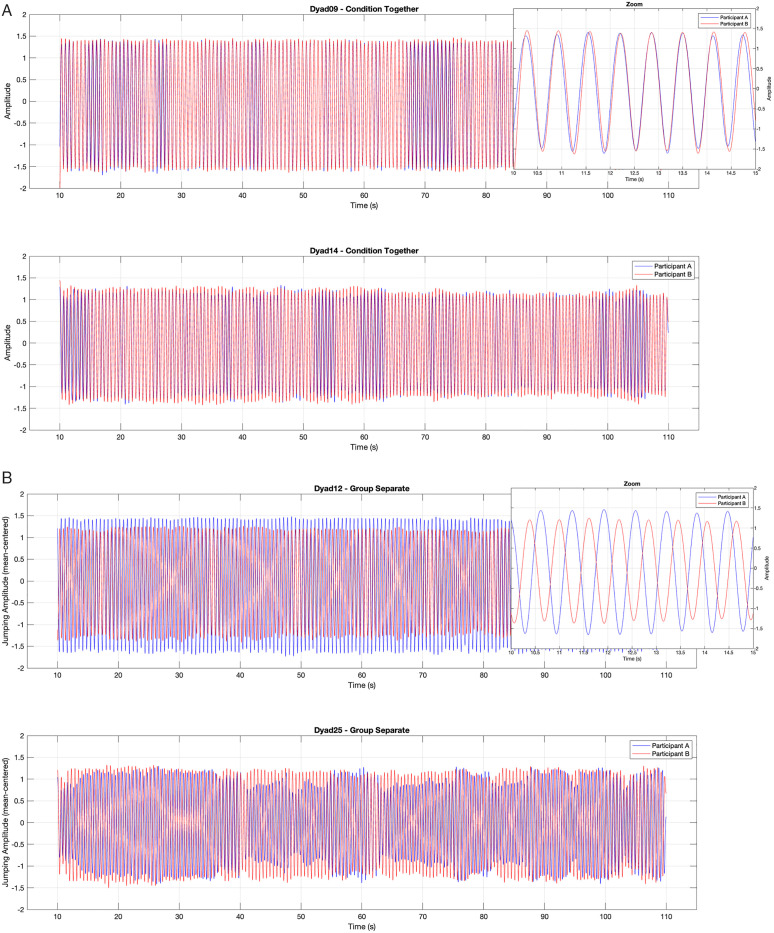
Examples of jumping synchrony trajectories. A) Example time-series from the two most strongly synchronized dyads (both group *together*) and B) the two most weakly synchronized dyads (both group *separate*). *Note*: The x-axis represents the time across the full jumping session (in seconds); the y-axis shows the amplitude of the mean-centered vertical movement, reflecting participants’ oscillatory jumping behavior.

Time-series plots of selected dyad jumping amplitudes reveal sinusoidal movement patterns, as well as frequency and phase shifts over time, distinguishing dyads with high versus low synchrony (Fig 1A and 1B). Descriptive data on jumping frequency showed that dyads in both groups had similar frequency patterns (see Table 1).

**Table 1:** Means and Standard Deviations of Jumping Frequency (Hz).

	Participant A		Participant B		Absolute Difference	
Group	*M*	*SD*	*M*	*SD*	*M*	*SD*
Together	1.68	0.13	1.72	0.11	0.07	0.09
Separate	1.68	0.11	1.68	0.13	0.10	0.10

**Fig 2:**
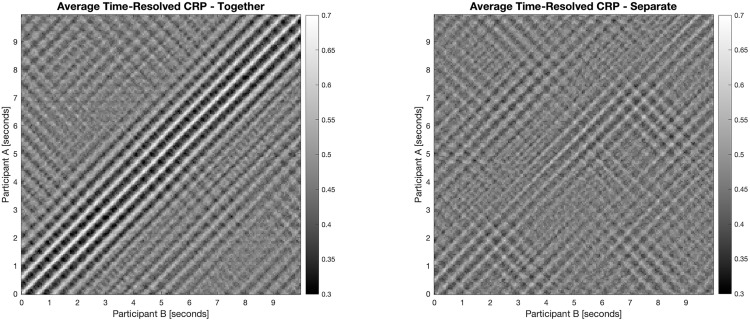
Cross-recurrence plots (CRPs) averaged across sliding windows and dyads within each group, shown for the group *together* (left) and *separate* (right). Lighter diagonal bands along the main (zero lag) diagonal indicate higher synchrony (i.e., greater movement similarity at lag zero). Axes represent time in seconds within the 10 second window; each pixel indicates recurrence between two time points—one from each dyad member’s movement time series.

The additional generated CRPs provide insights into time-resolved dynamics of behavioral synchrony by revealing how similar the participants’ movements were over time. The experimental group *together* displayed a pronounced diagonal line, indicating strong recurrent coordination patterns with consistent temporal structure. In contrast, the CRPs of the group *separate* lacked such structure, suggesting weaker or less consistent synchrony. These patterns imply a diagonal recurrence profile (DRP) with a sharp peak at lag zero in the group *together*, and a flatter DRP in the group *separate* (Fig 2). Consistent with the visual patterns, the group together showed higher values across all metrics compared to the group separate, indicating longer periods of synchronous dynamics and stronger coupling between participants. Although these differences did not reach conventional levels of statistical significance, the trend mirrors the CRP results, supporting the conclusion of more pronounced synchrony in the group together.

**Fig 3:**
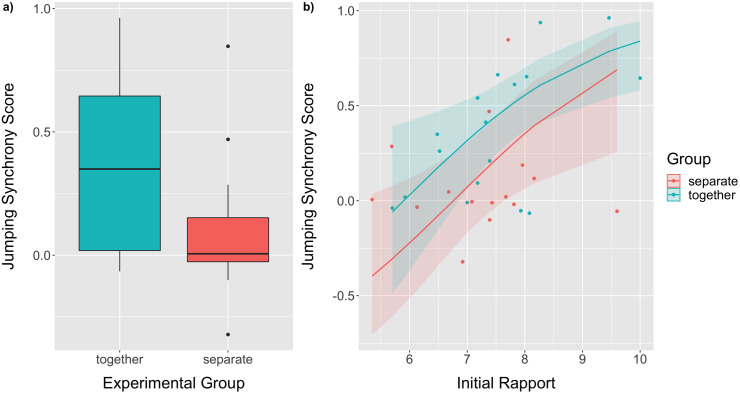
Group differences in jumping synchrony. A) Jumping synchrony scores in the experimental groups *together* (blue) and *separate* (together). The box plots represent medians (center horizontal lines), inter-quartile ranges (boxes), as well as minima and maxima (whiskers) and outliers (dots). B) The effect of the participants’ initial rapport (averaged across the two dyad members) on the dyad’s jumping synchrony score. The lines show the predicted values of the linear model with the predictors experimental group and initial rapport, while holding the non-significant predictor initial mood fixed at its mean. The shaded areas represent the pointwise 95% confidence intervals of the predicted values. Predicted values have been back-transformed to the original scale of the dependent variable (bounded between −1 and 1). The raw data are represented with dots.

As predicted, we found that the participants who had previously worked on the jigsaw puzzle together displayed more synchrony during jumping (**M* *= 0.364, *SD *= 0.366) than the participants who had worked on the jigsaw puzzles separately (**M* *= 0.095, *SD *= 0.274; **t* *= −2.410, **df* *= 30, **p* *= 0.022, *d* = −0.85; Fig 3A). The linear model that included experimental group as well as initial rapport and mood as factors revealed a significant regression for the jumping synchrony score (*R*^2^= 0.337, *F*(3,28) = 4.750, **p* *= 0.008). Initial rapport significantly predicted jumping synchrony score (Fig 3B), while experimental group and initial mood did not predict jumping synchrony (for detailed model results, see Table 2).

**Table 2:** Unstandardized (Beta) and standardized coefficients (β) of the linear model: predicting jumping synchrony from experimental group (together vs. separate), initial rapport, and mood. Beta, 95% confidence intervals (CI), and β have been back-transformed to the original scale of the dependent variable (bounded between −1 and 1). Standard errors (*SE*), *t*-values, *p*-values, and Cohen’s *f²* remain on the Fisher-transformed scale.

Variable	Beta	95% CI	β	*SE*	*t*	*p*	*f²*
(Intercept)	−0.706	[-0.977, 0.428]		0.653	−1.347	0.189	
Group	0.251	[-0.093, 0.541]	0.241	0.171	1.506	0.143	0.28
Rapport	0.289	[0.082, 0.473]	0.540	0.105	2.827	0.009	0.53
Mood	−0.149	[-0.359, 0.075]	−0.285	0.110	−1.364	0.184	0.26

When investigating how rapport and mood changed over time, we found that rapport increased significantly over time (*M*_before_ = 7.36, *SD* = 1.661; *M*_after_ = 8.16, *SD* = 1.432; Table 3). There was no effect of the experimental group or of the interaction between experimental group and measurement time on rapport. For mood, there was a significant main effect of measurement time and a significant interaction effect between experimental group and measurement time (Table 3). The experimental group had no effect on participants’ mood. When splitting the data by experimental group, we found that mood increased significantly after participants had worked on the puzzle together (*M*_before_ = 7.40, *SD* = 1.476; *M*_after_ = 8.41, *SD* = 1.388; **t* *= −5.462, **df* *= 33, *p*_*corr*_* *≤ 0.001, *d* = −0.94), but not after they had worked on the puzzle separately (*M*_before_ = 7.81, *SD* = 1.249; *M*_after_ = 8.01, *SD* = 1.341; **t* *= −0.922, **df* *= 32, *p*_*corr*_* *= 0.726, *d* = −0.16).

**Table 3:** Results of the two mixed ANOVAs: Change of rapport and mood in the experimental groups (*together* vs. *separate*) over time.

	Rapport			Mood		
Variable	*F*(1, 65)	*p*	η_G_^2^	*F*(1, 65)	*p*	η_G_^2^
Group	1.180	0.281	0.02	0.001	0.980	0.00
Time	47.788	<0.001	0.06	18.229	<0.001	0.05
Group * Time	0.466	0.497	0.00	8.228	0.006	0.02

## Discussion

The main goal of the current study was to investigate whether a cooperative interaction between two individuals induces greater interpersonal synchrony in a subsequent unrelated task. As predicted, we found that dyads who had collaboratively worked on a jigsaw puzzle exhibited more movement synchrony when later jumping on trampolines, than dyads that had worked individually. When we tested for the additional influence of the participants’ initial rapport and mood, the effect of the collaborative treatment was qualified by a stronger effect of initial rapport.

Previous research has shown that interpersonal synchrony emerges during collaborative interactions [20,22,24] and sometimes even endures beyond the period of collaboration [25]. Moreover, the degree of interpersonal coordination rises as the demands of the cooperative precision of the task increases [34]. One has to consider, though, that in set-ups that measure cooperation and synchrony simultaneously in the same task, coordinating movements in a synchronous way might just be the most efficient way for multiple individuals to achieve their goals. Allsop et al. [24] found that two participants that had to simultaneously move balls from one container to another synchronized their movements more than expected by chance, both when they were instructed to cooperate in the task and when they were instructed to compete. Nevertheless, their movements were more synchronized during cooperation than during competition [24]. Even when measuring synchrony after the period during which participants had been instructed to cooperate, but continuing to measure it in the same task might promote synchrony due to lingering entrainment or facilitation effects [25]. By using two different tasks as treatment and test, which differed strongly in their structuring, affordances, and movement patterns, our results provide even stronger support for the purported bi-directional association between cooperation and interpersonal synchrony [11].

Nevertheless, our results also highlight the importance of rapport for interpersonal synchrony and show that initial feelings of rapport (or the lack thereof) can outweigh the impact of collaborative interactions. Initial rapport between the interacting partners was, in fact, the strongest predictor of their jumping synchrony. Interestingly, Valdesolo and colleagues [9] found a different pattern when examining the relationship between synchrony, cooperative abilities, and social traits with the reverse experimental set-up, namely investigating the effect of a synchrony condition (rocking in chairs side-by-side) vs. an asynchrony condition (rocking in chairs back-to-back) on the completion of a coordinative joint task. They found that subsequent cooperative abilities were positively affected by the perceptual sensitivity elicited by the synchrony condition, but not by measures of social closeness. The authors conclude that a joint action task that necessitates the calibration of perceptual and motor skills profits from individuals’ enhanced sensitivity towards another person experienced during interpersonal synchrony, and can function independently of social bonding mechanisms [9]. Our results, however, corroborate previous findings that a person’s positive orientation towards others and the rapport between two individuals enhances interpersonal synchrony [3,14,15,19]. To understand these seemingly contradictory results, it is important to distinguish between the different tasks in both experiments. Solving a jigsaw puzzle requires both verbal and nonverbal communication as well as social collaboration, which may be enhanced by high rapport. This, in turn, influences synchrony in spontaneous movement coordination, such as trampoline jumping. Conversely, synchronous swinging in rocking chairs, as it was used in the study by Valdesolo and colleagues [9], is a rhythmic movement that does not require verbal or strategic interaction. The subsequent cooperative motor task may have involved a different type of coordination, relying less on social interaction—such as verbal and nonverbal communication—and more on perceptual sensitivity and individual motor coordination, which appears to be less affected by social bonding mechanisms [9,25]. Future studies investigating cooperation tasks with varying demands for communication and motor coordination could provide deeper insights into the effects of perceptual sensitivity and social bonding on interpersonal synchrony.

In our set-up it is difficult to disentangle whether the positive effect of initial rapport was caused by the participants’ inherent attitudes towards others or by an initial liking between the two interaction partners. Although all dyad members were strangers and no specific instructions were given about the collaborative nature of the treatment phase, the two participants were together in the same room when the experimenter greeted them and explained the general procedure to them. Only after this initial phase, they were seated at two separate desks to score their expected rapport with the other person. It is possible that this brief interaction was sufficient to establish a certain degree of rapport with the previously unknown person [3]. Alternatively, prosocial-oriented participants might have had a general tendency to expect higher levels of rapport with another person, irrespective of the first impression, which might have in turn affected their initial rapport scores and their later synchronization. To disentangle these effects, future studies should use questionnaires that probe the participants’ other-related orientations and expected rapport before the participants meet for the first time. They should then use a separate questionnaire to assess the strength of rapport after the interaction partners are allowed to meet and interact for a short period and test whether their rapport changed after getting a first impression of the other person.

When investigating changes in rapport in the current study, we found that the participants’ rapport increased from the initial rating before the experiment to the second rating at the very end of the experiment, irrespective of whether the participants had worked on the puzzle collaboratively or individually. This is in contrast to a previous study, which investigated changes in rapport after a social interaction and found rapport ratings to depend on the previous degree of synchronization during the task [40]. Whereas Bernieri [40] measured synchrony during a competitive teacher-student task, however, the participants in the current experiment engaged in cooperative (i.e., collaborative puzzle task) and/or neutral, non-competitive tasks (i.e., individual puzzle task; jumping task). Rapport between two individuals has been described as a dynamic structure, which initially develops through simple positivity and mutual friendliness. Only in continued interactions do individuals start to evaluate the degree of smoothness of their interaction and adjust their feelings of rapport [41]. It is possible that the participants in both experimental groups of the current study perceived their dyad partner as generally friendly and thus experienced an increased level of rapport throughout the experiment, irrespective of the specific interactions in the treatment phase. During the competitive interactions investigated by Bernieri [40], on the other hand, it is likely that the participants did not experience much initial positivity and friendliness and that rapport in that study depended much more on the actual quality of the interaction. Put together, any positive and neutral, but not negative social interactions appear to result in the emergence of a basic level of rapport, which was likely the case in the current study. Importantly, the effect of an initial feeling of rapport on synchronization levels accounts for spontaneous and unintentional synchronization forms, like walking in step or clapping hands in an audience, and does not necessarily apply to intentional or even entrained synchrony, like a choreographed dance or a marching army.

Differently from the indiscriminate increase of rapport, for mood, we found the predicted effect that mood increased significantly only for the participants who had worked on the puzzle together, but not for the participants that had worked on the puzzle individually. This finding supports previous research that found that positive affect increases after an explicitly social interaction, such as having lunch with a newly acquainted person [42]. The enjoyment of the collaborative puzzle task and the resulting increased mood thus seem like good candidates for the mechanism underlying the higher levels of interpersonal synchrony after working on the puzzle together. However, initial mood rated before the experiment was no good predictor of later synchronization. Miles et al. [14] found that mood assessed in a general sense was not related to the subsequent occurrence of interpersonal synchrony. The authors argue that self-assessed mood represents a rather explicit state, which does not strongly impact an experimental task [14]. We assume that participants rated their mood at the beginning of the experiment as a rather explicit state that was based on their day-to-day experiences before they arrived at the lab. This resulted in a range of initial mood ratings across the whole sample, which did not strongly affect later interactions between the dyad members. Participation in the experiment, on the other hand, was likely a quite unique experience for the participants, allowing a much more targeted influence on their mood, which resulted in a stronger increase in the participants’ mood when working on the jigsaw puzzle together than individually. To further investigate how positive mood influences interpersonal synchrony, one could compare collaborative tasks designed to enhance participants’ mood with tasks that either have no effect or decrease mood, and then assess their impact on subsequent synchronization. One could additionally contrast these effects with treatments that are non-social, but are designed to increase the participants’ mood. In light of the complex interrelations between mood, rapport, and collaborative interactions, more research, ideally with larger sample sizes, will be necessary to quantify the true impact of each of these factors on interpersonal synchrony.

## Limitations

One limitation of this study was the relatively small sample size. We based our sample size estimation on previous studies on interpersonal synchrony (e.g., [24,29,30]), which demonstrated large effect sizes when testing the association between interpersonal synchrony, cooperation, and affiliation. A recent meta-analysis on the effect of synchrony on social outcomes, however, showed that effect sizes varied considerably across studies [8,13], illustrating that researchers are often confronted with a degree of uncertainty when choosing effect sizes and estimating sample sizes for novel experimental set-ups such as the one used in the current study [43]. Future research should take advantage of novel approaches, such as bias- and uncertainty-corrected sample size estimation [43] or Bayesian sequential testing and Bayes factor design analysis [44], to tackle this important issue.

Another limitation of this study is the possibility of additional influencing factors on the jumping synchrony. Besides psychological predispositions like rapport and mood, physical conditions of the participants could have affected synchronization rates. As shown by Richardson and colleagues [45], the eigenfrequency of the chair, experimentally manipulated by attaching mass to the chair’s base, impacts the degree of synchronization that occurs spontaneously between individuals [46]. While the size of the trampolines was held constant in both conditions, data about the fitness, body mass, and height of the participants were not collected and could therefore not be taken into account. Although participants represented a relatively homogenous group, consisting mostly of students with fairly good fitness levels (median age: 23.5 years), follow-up studies should record and analyze additional individual data.

In the current study design, we cannot rule out that cognitive dissonance induced by the transition from a collaborative puzzle task to an unrelated trampoline activity may have affected synchrony to some extent. Importantly, however, both groups received the same information and successive order of tasks and any task-related cognitive dissonance would have likely affected both groups equally. Nevertheless, future research should explore whether cognitive dissonance effects interact with prior cooperative activities or social interactions and thus influence synchrony.

And lastly, while behavioral synchrony of everyday activities such as walking or clapping hands has been documented, the simplicity of the current synchrony task allows only for an initial examination of the link between collaborative work and movement coordination. Future research may want to transfer this relatively artificial set-up into a more ecologically relevant context, such as freestyle dancing, running, or walking.

Overall, the results should be interpreted with some caution until confirmed by studies with larger samples or refined analytical approaches, as the significant findings from the cross-correlation analysis were not statistically supported by the CRQA metrics. Nevertheless, these metrics showed a consistent trend in the same direction, and the CRPs indicated a clear differentiating pattern.

## Conclusion

Despite its limitations, the present study offers valuable insights into how cooperative interactions may influence subsequent interpersonal synchrony. By showing that participants who collaborated on a puzzle task exhibited more movement synchrony in a later, unrelated activity, we provide initial evidence that the effects of cooperation can carry over into other domains of interaction [47]. Furthermore, the level of synchrony is closely linked to the social connection between interacting partners [2], which is reflected in our finding that initial feelings of rapport influenced synchronization rates within pairs. The current findings highlight the crucial role of collaborative interactions in fostering spontaneous interpersonal synchrony in a repetitive movement task. In a more general sense, this form of movement coordination has repeatedly been explained through the dynamics of coupled oscillators [4], aligning with the idea of functional synchronization, where basic elements, e.g., neural activity, movements, and thoughts, dynamically coordinate to create a unified functional unit relevant to the task. Together, our findings add a further puzzle piece to the growing body of research suggesting that interpersonal synchrony is shaped not only by shared activities but also by the quality of social relationships. Future studies are needed to explore in detail how different forms of interaction and social context contribute to the emergence of synchrony in real-world settings.
